# Supplementary material for: Instrument-free detection of polyphenols with a thread-based analytical device
Source: R Soc Open Sci. 2020 Mar 11;7(3):192130. doi: 10.1098/rsos.192130 (PMC7137958; doi:10.1098/rsos.192130)
Supplement: Electronic Supplementary Material [file rsos192130supp1.docx]

**Electronic Supplementary Material**

**Instrument-free detection of polyphenols with a thread-based analytical device**

Jiahong Song,† Zhuang Ouyang,† Wei Lu and Longfei Cai*

*School of Chemistry and Environmental Engineering, Hanshan Normal University, Chaozhou, Guangdong 521041, China.*

**1. The length data with different Fe^2+^ Concentration**

Table 1. The length of colored band formed on the threads with 0.2, 0.4 and 0.8 g L^-1^ of Fe^2+^ (three replica runs were performed for each concentration).

|  | 0.2 g L^-1^ | 0.4 g L^-1^ | 0.8 g L^-1^ |
| --- | --- | --- | --- |
| 1 | 24 mm | 21 mm | 16 mm |
| 2 | 27 mm | 25 mm | 19 mm |
| 3 | 25 mm | 22 mm | 19 mm |

**2. The data obtained with different pH**

Table 2. The length of colored band obtained by varying pH of chromogenic reagents solution (three replica runs were performed for each pH).

|  | pH=2 | pH=3 | pH=4 | pH=5 |
| --- | --- | --- | --- | --- |
| 1 | 30 mm | 22 mm | 22 mm | 22 mm |
| 2 | 32 mm | 23 mm | 21 mm | 23 mm |
| 3 | 32 mm | 23 mm | 21 mm | 21 mm |

**3. The data obtained with different polyphenol concentration**

Table 3. The length of colored band obtained with different concentrations of polyphenols (three replica runs were performed for each concentration).

|  | 0.1 g L^-1^ | 0.2 g L^-1^ | 0.4 g L^-1^ | 0.6 g L^-1^ | 0.8 g L^-1^ |
| --- | --- | --- | --- | --- | --- |
| 1 | 13 mm | 17 mm | 22 mm | 26 mm | 28 mm |
| 2 | 14 mm | 18 mm | 21 mm | 26 mm | 30 mm |
| 3 | 14 mm | 17 mm | 22 mm | 25 mm | 30 mm |

**4. The data used for calculation of detection limit**

Table 4. The length of colored band from 11 replica assays of 0.05 g L^-1^ polyphenol solution.

| Operation | 1 | 2 | 3 | 4 | 5 | 6 | 7 | 8 | 9 | 10 | 11 |
| --- | --- | --- | --- | --- | --- | --- | --- | --- | --- | --- | --- |
| Length (mm) | 9 | 10 | 11 | 10 | 10 | 10 | 10 | 10 | 11 | 11 | 9 |

**5. The data obtained from the study of effect of foreign species (glucose, protein, caffeine, ascorbic acid and amino acids)**

Table 5. The length of colored band obtained with solution containing 0.4 g L^-1^ polyphenols and solution containing 0.4 g L^-1^ polyphenols and 0.4 g L^-1^ glucose (three replica runs were performed for each solution).

|  | 0.4 g L^-1^ polyphenols | 0.4 g L^-1^ polyphenols + 0.4 g L^-1^ glucose |
| --- | --- | --- |
| 1 | 21 mm | 21 mm |
| 2 | 22 mm | 22 mm |
| 3 | 20 mm | 19 mm |

Table 6. The length of colored band obtained with solution containing 0.4 g L^-1^ polyphenols and solution containing 0.4 g L^-1^ polyphenols and 0.4 g L^-1^ protein (three replica runs were performed for each solution).

|  | 0.4 g L^-1^ polyphenols | 0.4 g L^-1^ polyphenols + 0.4 g L^-1^ proteins |
| --- | --- | --- |
| 1 | 21 mm | 21 mm |
| 2 | 21 mm | 22 mm |
| 3 | 23 mm | 21 mm |

Table 7. The length of colored band obtained with solution containing 0.4 g L^-1^ polyphenols and solution containing 0.4 g L^-1^ polyphenols and 0.4 g L^-1^ caffeine (three replica runs were performed for each solution).

|  | 0.4 g L^-1^ polyphenols | 0.4 g L^-1^ polyphenols + 0.4 g L^-1^ caffeine |
| --- | --- | --- |
| 1 | 23 mm | 22 mm |
| 2 | 22 mm | 23 mm |
| 3 | 23 mm | 22 mm |

Table 8. The length of colored band obtained with solution containing 0.4 g L^-1^ polyphenols and solution containing 0.4 g L^-1^ polyphenols and 0.06 g L^-1^ ascorbic acid (three replica runs were performed for each solution).

|  | 0.4 g L^-1^ polyphenols | 0.4 g L^-1^ polyphenols + 0.06 g L^-1^ ascorbic acid |
| --- | --- | --- |
| 1 | 19 mm | 21 mm |
| 2 | 20 mm | 18 mm |
| 3 | 21 mm | 21 mm |

Table 9. The length of colored band obtained with solution containing 0.4 g L^-1^ polyphenols and solution containing 0.4 g L^-1^ polyphenols and 0.2 g L^-1^ amino acids (three replica runs were performed for each solution).

|  | 0.4 g L^-1^ polyphenols | 0.4 g L^-1^ polyphenols + 0.2 g L^-1^ amino acids |
| --- | --- | --- |
| 1 | 22 mm | 21 mm |
| 2 | 22 mm | 21 mm |
| 3 | 20 mm | 22 mm |

**6. The data of real sample analysis with thread-based devices**

Table10. The length of colored band resulted from three replica assays of the sample which was four-folded diluted.

| Operation | 1 | 2 | 3 |
| --- | --- | --- | --- |
| Length | 20 mm | 23 mm | 33 mm |

7. **Data with a standard method (spectrophotometric method)**

Table 11. Absorbance obtained with different polyphenols concentrations (three replica runs were performed for each concentration).

|  | 0.2 g L^-1^ | 0.4 g L^-1^ | 0.6 g L^-1^ | 0.8 g L^-1^ | 1.0 g L^-1^ |
| --- | --- | --- | --- | --- | --- |
| 1 | 0.094 | 0.152 | 0.219 | 0.287 | 0.346 |
| 2 | 0.095 | 0.153 | 0.221 | 0.287 | 0.349 |
| 3 | 0.091 | 0.154 | 0.220 | 0.288 | 0.347 |

Table 12. The absorbance obtained from three replica assays of the sample which was two-folded diluted.

|  | 1 | 2 | 3 |
| --- | --- | --- | --- |
| Absorbance | 0.322 | 0.322 | 0.324 |
